# Supplementary material for: Unveiling the RKIP and EGFR Inverse Relationship in Solid Tumors: A Case Study in Cervical Cancer
Source: Cancers (Basel). 2024 Jun 10;16(12):2182. doi: 10.3390/cancers16122182 (PMC11202200; doi:10.3390/cancers16122182)
Supplement: Supplementary file 1 [file cancers-16-02182-s001.zip › Legends_Supp Figures.pdf]

Figure S1. Clinical impact of RKIP / EGFR negative feedback - *in silico* analysis. (A) mRNA expression level and survival analysis of EGFR and PEBP1 across the 30 TCGA cancer types (GEPIA2 - http://gepia2.cancer-pku.cn, last accessed on 14 April 2024). Heatmap of hazard ratios (HR) illustrating cancer-EGFR/PEBP1 pairs with altered prognosis. The median was selected as the suitable expression threshold for splitting the high-expression (red) and low-expression (blue) cohorts and the HR was calculated based on the Cox PH Model. The intensity of color indicates the value of HR and the bounding box around the tiles represents the statistically significant cancer types (p<0.05). **(B)** GEPIA2 online Kaplan-Meier survival analysis was conducted with high (red) and low (blue) mRNA expression of PEBP1 and EGFR genes and their associations with overall survival in cervical squamous cell carcinoma (CESC). Logrank test <0.05 is considered as statistically significant.

Figure S2: Analysis of 1739 different cell lines available in Cancer Cell Line Encyclopedia (Broad, 2019) on CBioPortal. (A) Plot of the data regarding the PEBP1 correlation with EGFR at mRNA level (log RNA Seq V2 RSEM) in human cell lines. **(B)** Plot of the data regarding the PEBP1 correlation with EGFR at the protein level (z-scores of protein abundance ratios relative to bridge-sample) in human cell lines. Spearman's correlation coefficients range from -1 to +1. Pearson p value <0.05 is considered statistically significant.

Figure S3: Analysis of RKIP expression after EGFR pharmacological modulation. A) Western blot analysis of p-EGFR (Tyr1068), EGFR, and RKIP protein expression in SW756 cell line treated with increasing concentrations of Erlotinib (ER) for 2 hours, followed by 15 minutes of treatment with 10ng/ml of EGF. B) Analysis of EGFR and RKIP mRNA expression levels, done by qPCR in all cell lines treated for 2 hours with ER, at 2.5 µM, followed by 15 minutes of treatment with 10ng/ml of EGF, same conditions as presented in the figure 3F at the main text. The results were calibrated to β-Actin, used as a reference gene (N=3).

Table S1: Correlation analysis between RKIP and HER receptors expression in the 30 TCGA PanCancer Atlas Databases regarding solid tumors.

Table S2: Correlation analysis between RKIP and EGFR expression in the 30 TCGA PanCancer Atlas Databases regarding solid tumors.

Table S3: Correlation between RKIP and HER receptors overexpression in adenocarcinomas.

Table S4: Expression analysis of RKIP according to cervical cancer adenocarcinoma patient's clinical features.
