# Supplementary material for: Unveiling the RKIP and EGFR Inverse Relationship in Solid Tumors: A Case Study in Cervical Cancer
Source: Cancers (Basel). 2024 Jun 10;16(12):2182. doi: 10.3390/cancers16122182 (PMC11202200; doi:10.3390/cancers16122182)
Supplement: Supplementary file 1 [file cancers-16-02182-s001.zip › Supplementary Data_Western Blots.pdf]

Supplementary data

Original Western Blots (WB)

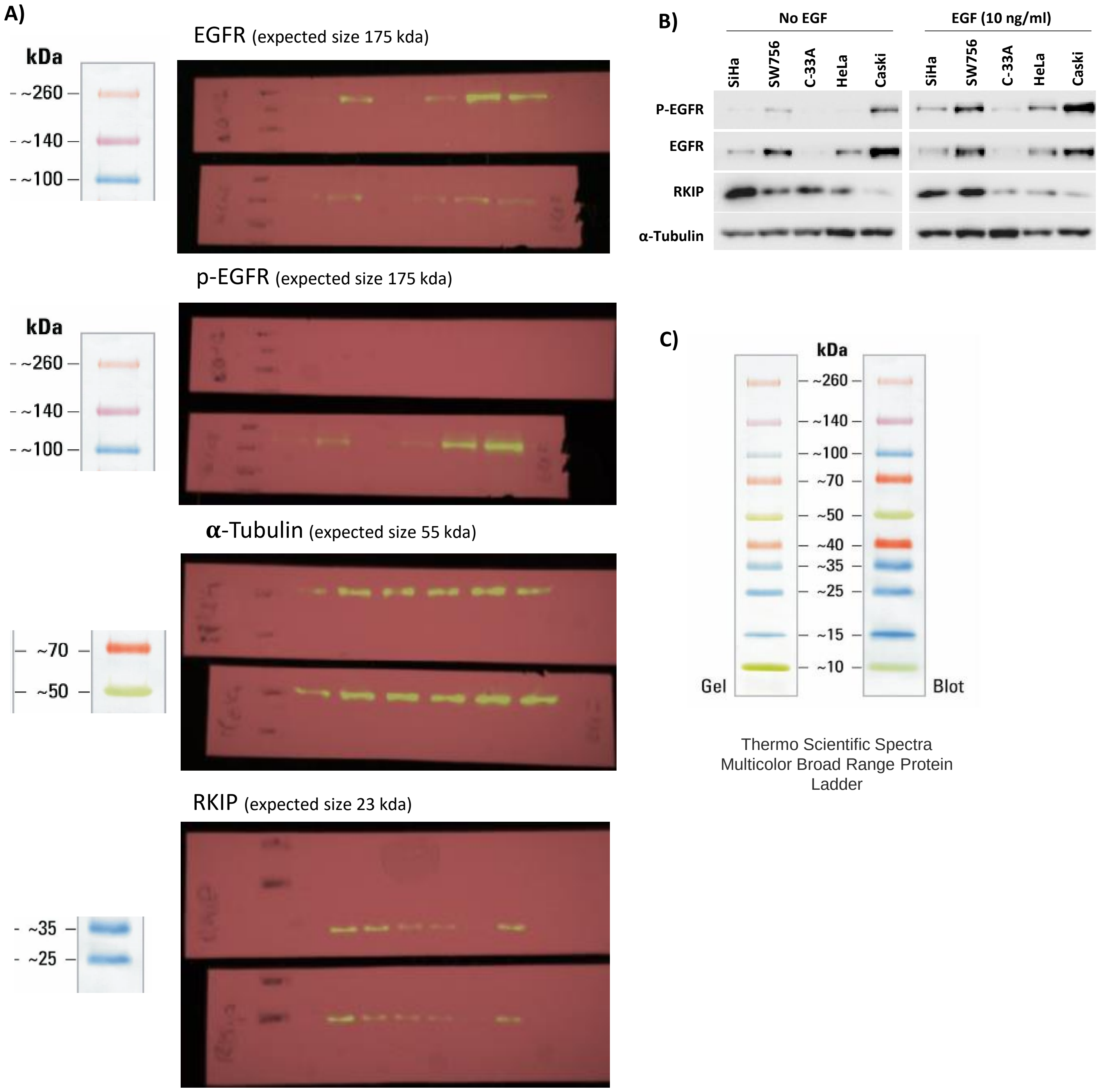

Figure: Original WB images with membrane merges, containing the protein marker for the paper figure 3A and C. All the antibodies used in the entire paper were previously tested for specificity and already published before in other papers of the group [1,2]. Taking advantage of this knowledge and to avoid unnecessary spending of money and biological material, for all the experiments the membranes were cut accordingly to the expected sizes of the proteins and based on the protein marker chosen **(C).** As example, for the Figure 3A and C of the paper **(B)**, we were able to recover the original images with membrane merges as a prove of specificity of our antibodies **(A).** The samples were ran in the same order as (B), and, as it can be observed, for comparison studies we ran the two conditions at the same time, and revealed the membranes for each antibody together. Also, in the merges with the membrane, the weaker bands are imperceptible, but appeared in the original revelation as it can be seen in the next figures. We always do the membrane merges in each revelation, but we rarely save it. Thus, Figure 3A and C was the only one for which we saved the merges, but in here are represented all the antibodies we used in the entire paper.

Figure 3A and 3C

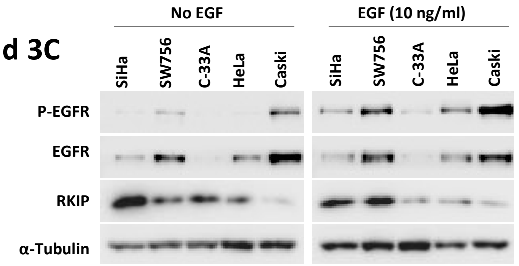

Original Revalation images

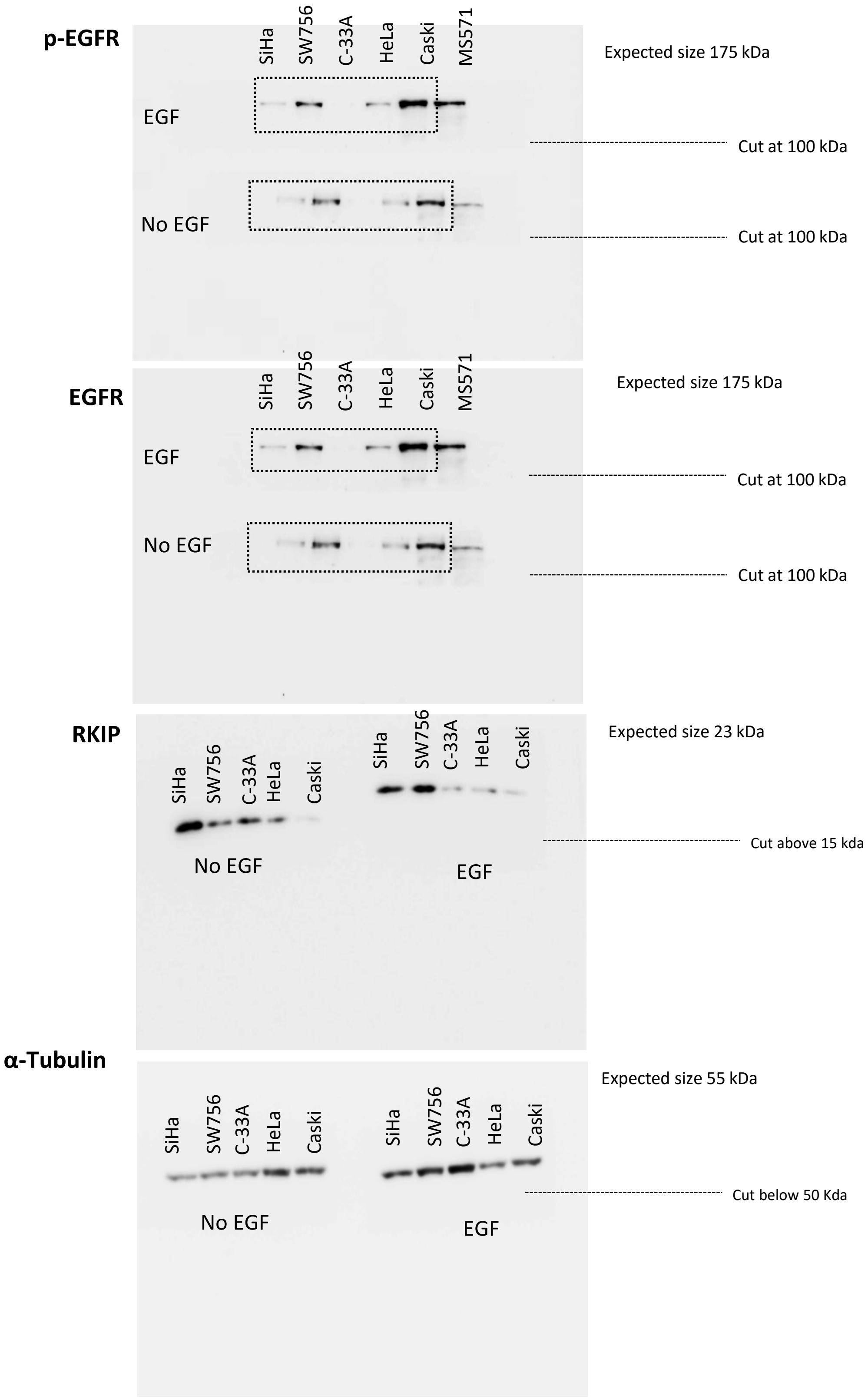

Figure: Original WB revelation images for each antibody presented in Figure 3A and C. In these experiments the membranes were cut as described in the pictures accordingly with the expected, and known sizes, and incubated with each antibody. The bands shown in Figure 3A, regarding pEGFR and EGFR, are the ones marked with a black square, because were revealed with other samples that were not included in the paper. All antibodies for the conditions EGF and No EGF, were revealed together for comparison.

Figure 3E

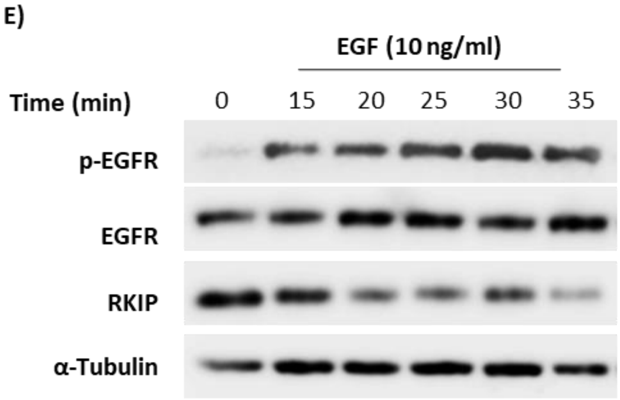

Original Revelation images

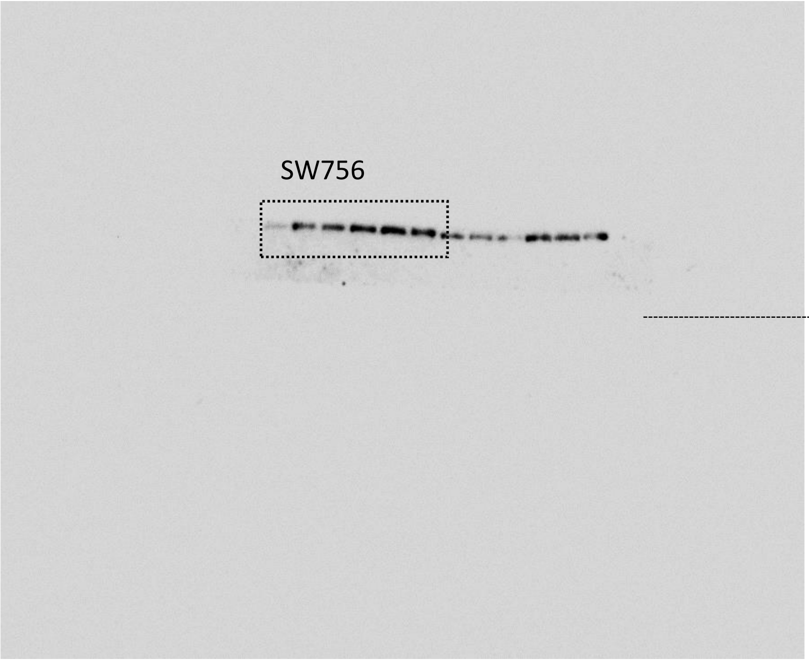

P-EGFR

Expected size 175 kDa

Cut at 100 kDa

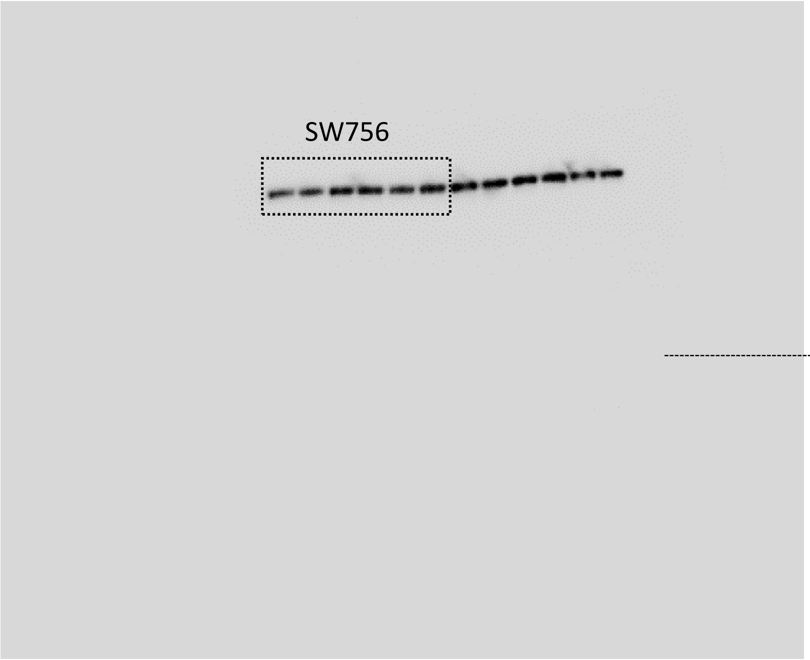

EGFR

Expected size 175 kDa

Cut at 100 kDa

(Continuation in the next page)

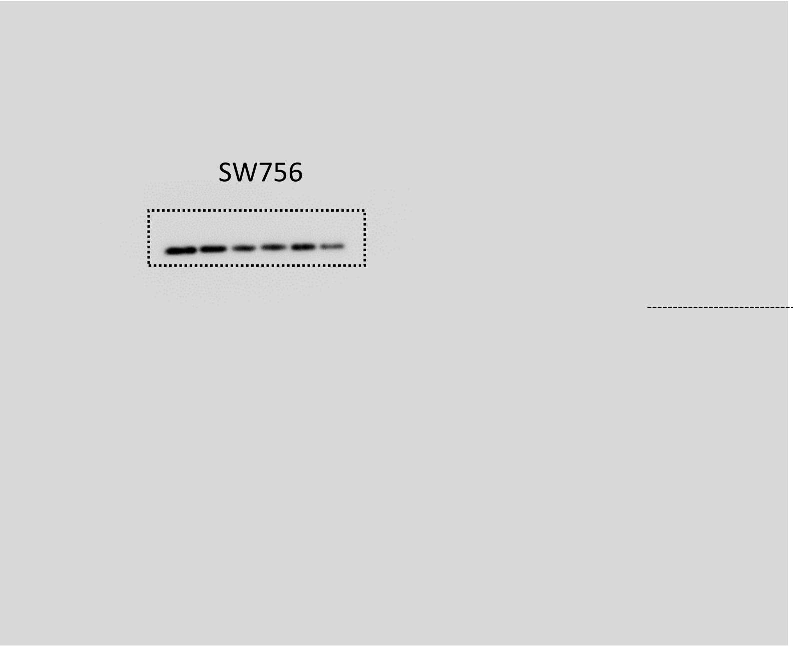

RKIP

Expected size 23 kDa

Cut above 15 kda

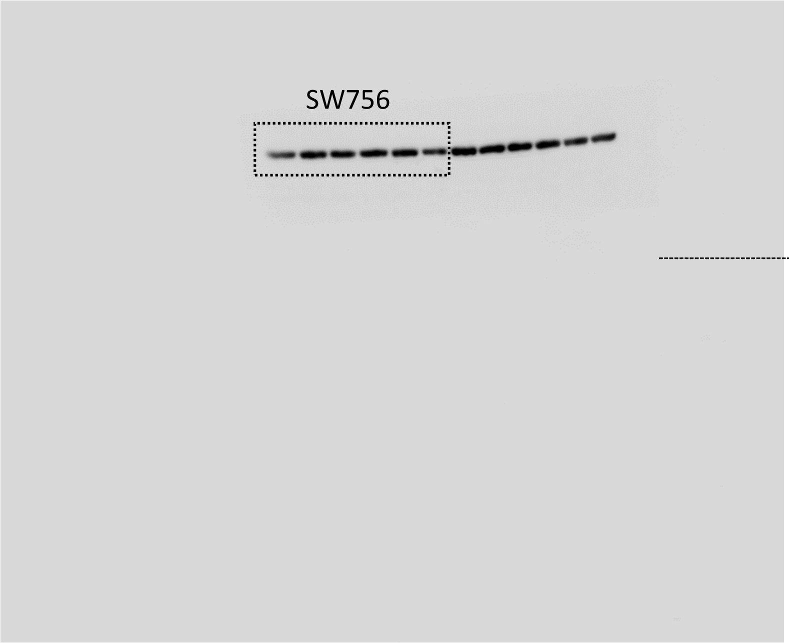

α-Tubulin

Expected size 55 kDa

Cut below 50 Kda

Figure: Original WB revelation images for each antibody presented in Figure 3E. In these experiments the membranes were cut as described in the pictures accordingly with the expected, and known sizes, and incubated with each antibody. The bands shown in Figure 3E are the ones marked with a black square, because were revealed with other samples that were not included in the paper.

Figure 3F

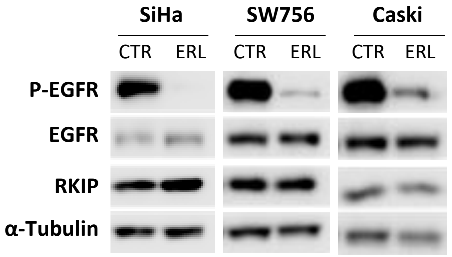

Original Revelation images

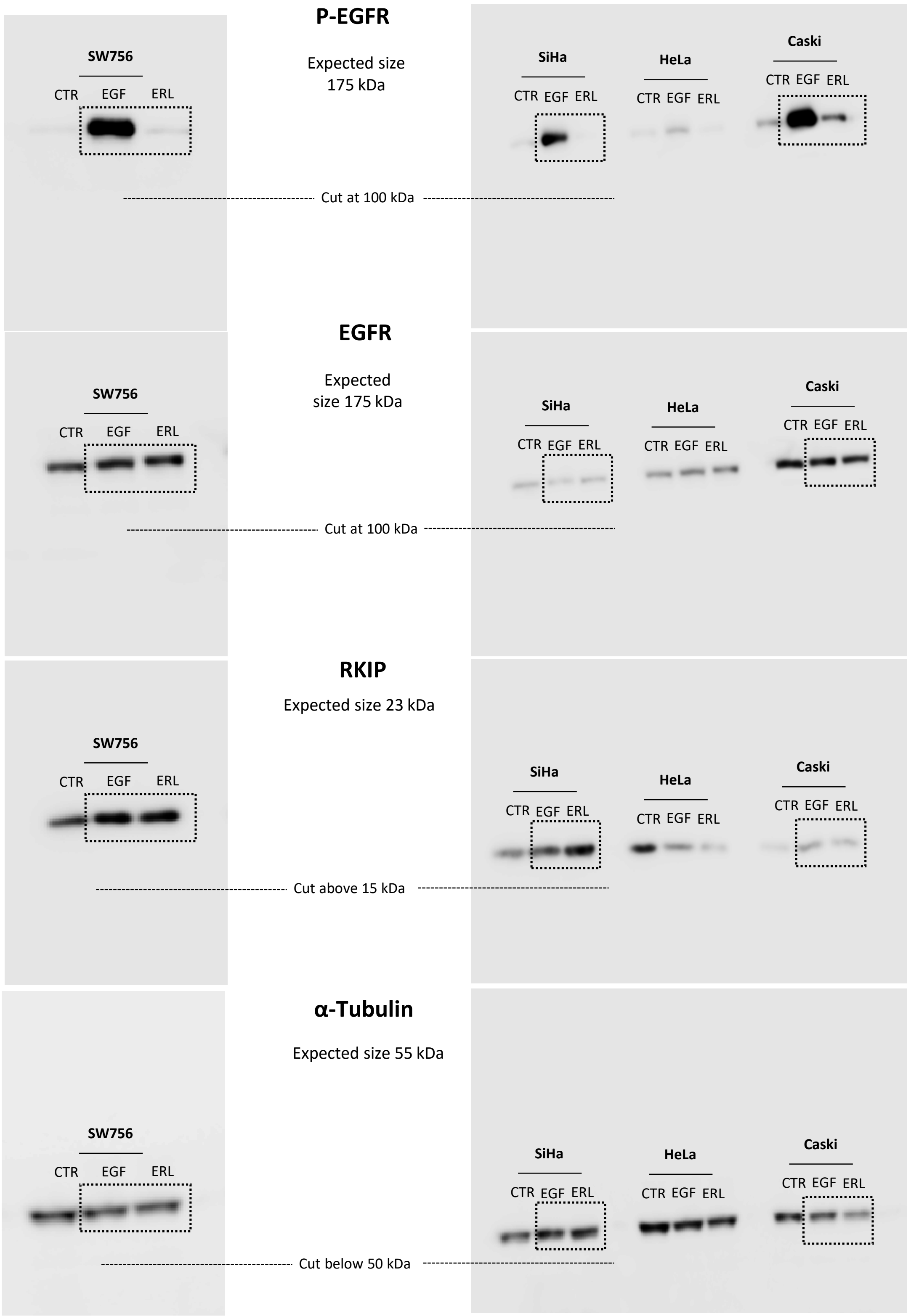

Figure: Original WB revelation images for each antibody presented in Figure 3B. In these experiments the membranes were cut as described in the pictures accordingly with the expected, and known sizes, and incubated with each antibody. The bands shown in Figure 3F are the ones marked with a black square, because were revealed with other samples that were not included in the paper and also with an internal technical CTR for us, that is not the one of the paper, being EGF condition our truly CTR in these experiments. Since we were unable to run all the cell lines in the same WB, SW756 was revealed in other membrane, which in that doesn't impact in the final results interpretation.

Figure 4A and B)

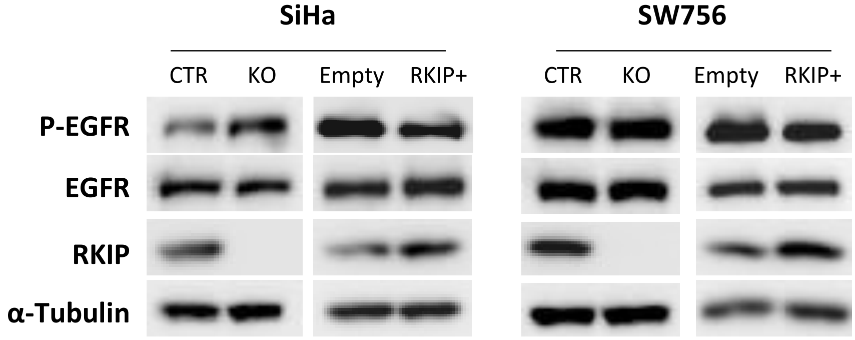

Original Revelation images

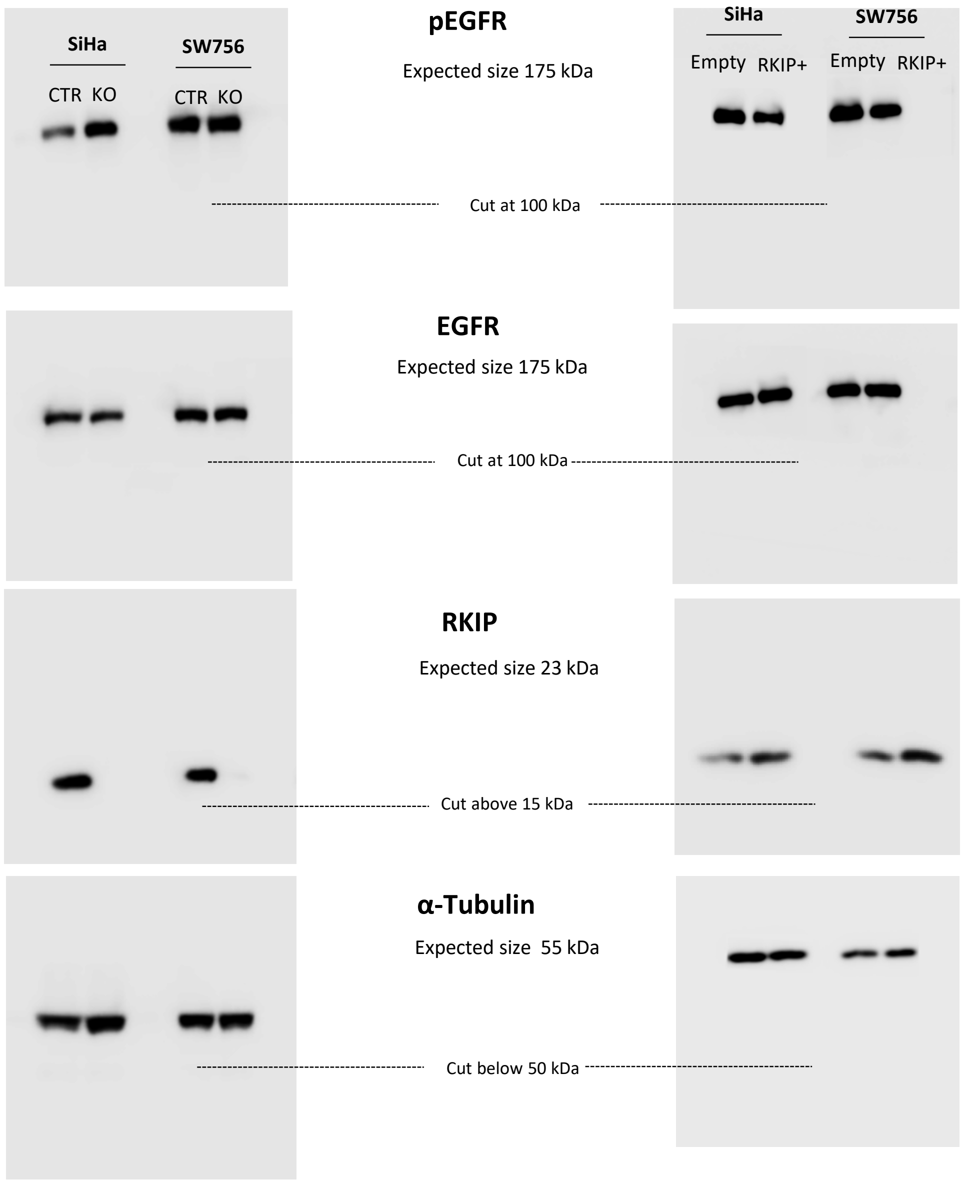

Figure: Original WB revelation images for each antibody presented in Figure 4A and B. In these experiments the membranes were cut as described in the pictures accordingly with the expected, and known sizes, and incubated with each antibody. The membranes from SiHa and SW756 cell line with RKIP knockout and overexpression were revealed separately to avoid losing signal from any band given the different expression levels in the two conditions.

FigureS3A)

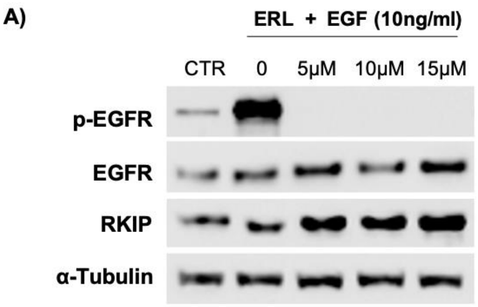

Original Revelation images

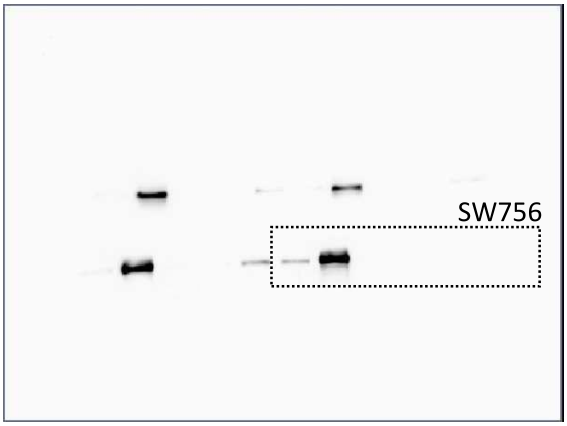

pEGFR

Expected size 175 kDa

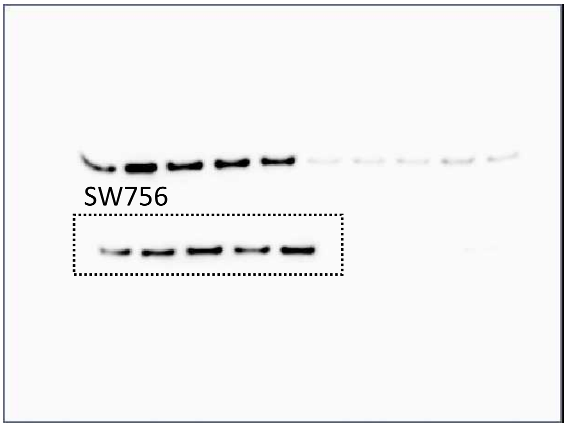

EGFR

Expected size 175 kDa

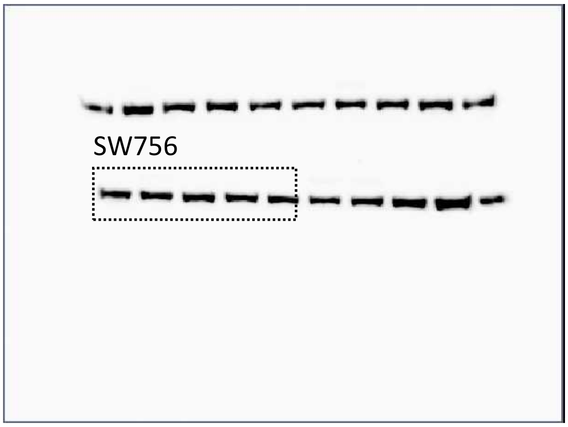

α-Tubulin

Expected size 55 kDa

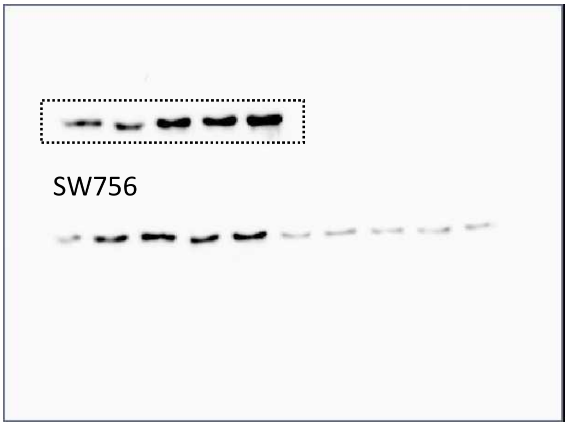

RKIP

Expected size 23 kDa

Figure: Original WB revelation images for each antibody presented in supplementar Figure 3E. In these experiments the membranes were cut as described in the pictures accordingly with the expected, and known sizes, and incubated with each antibody. The bands shown in Figure 3E are the ones marked with a black square, because were revealed with other samples that were not included in the paper.

References

1.Martinho, O., et al., *RKIP inhibition in cervical cancer is associated with higher tumor aggressive behavior and resistance to cisplatin therapy.* PLoS One, 2013. **8**(3): p. e59104.

2.Martinho, O., et al., *HER Family Receptors are Important Theranostic Biomarkers for Cervical Cancer: Blocking Glucose Metabolism Enhances the Therapeutic Effect of HER Inhibitors.* Theranostics, 2017. **7**(3): p. 717-732.
