# Supplementary figures and images for: Unveiling the RKIP and EGFR Inverse Relationship in Solid Tumors: A Case Study in Cervical Cancer
Source: Cancers (Basel). 2024 Jun 10;16(12):2182. doi: 10.3390/cancers16122182 (PMC11202200; doi:10.3390/cancers16122182)

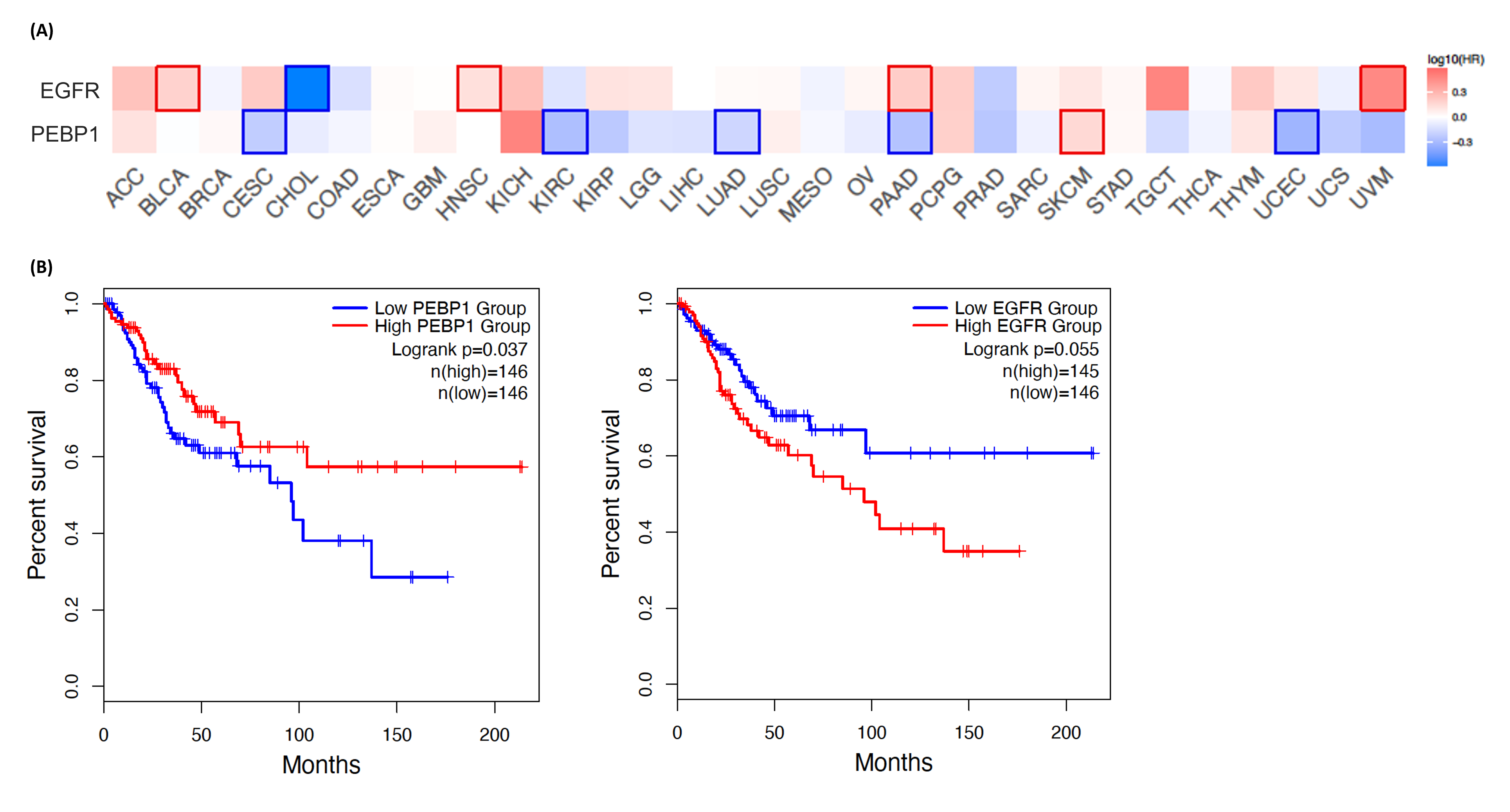

Supplement: Supplementary file 1 [file cancers-16-02182-s001.zip › FigureS1.png]

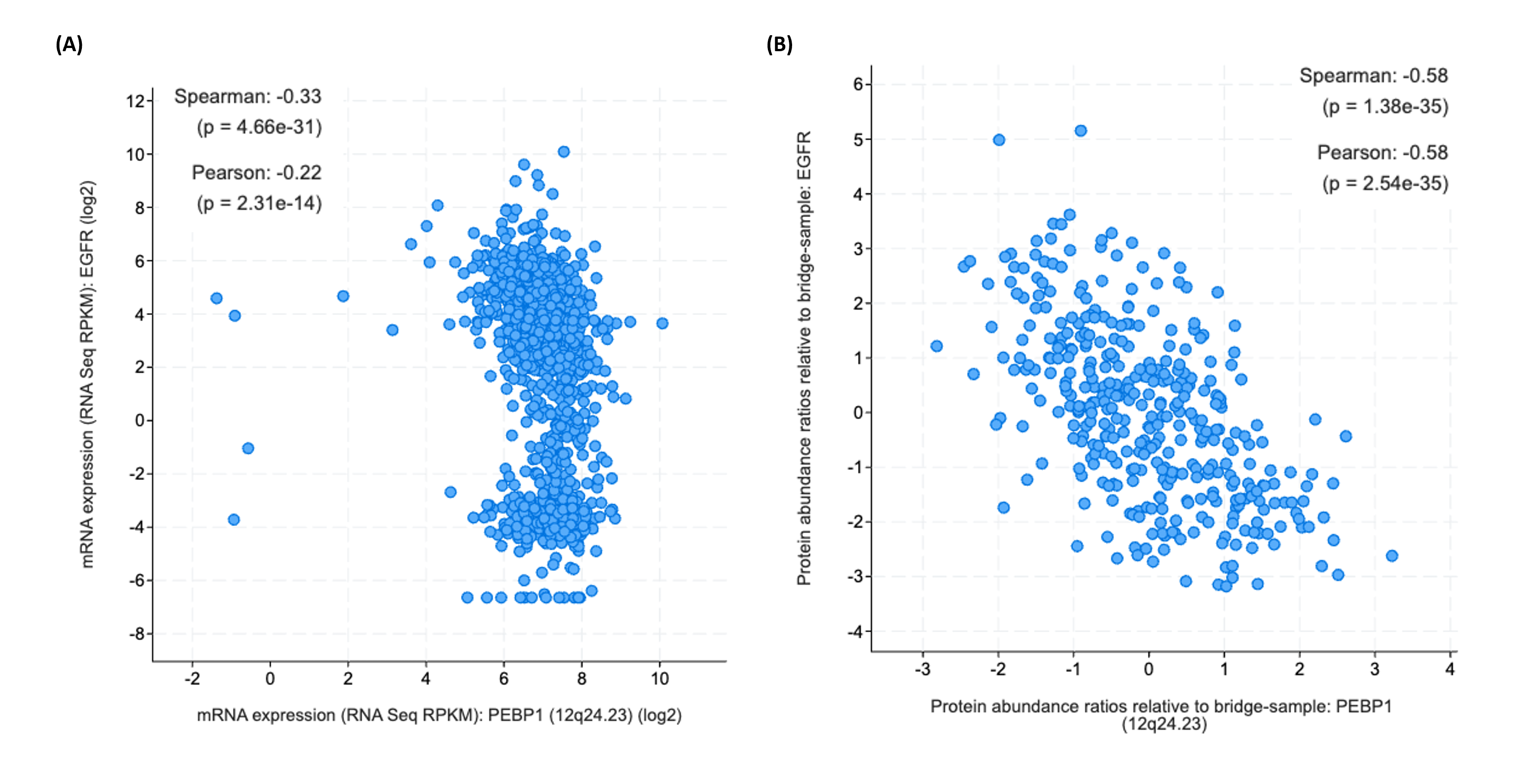

Supplement: Supplementary file 1 [file cancers-16-02182-s001.zip › FigureS2.png]

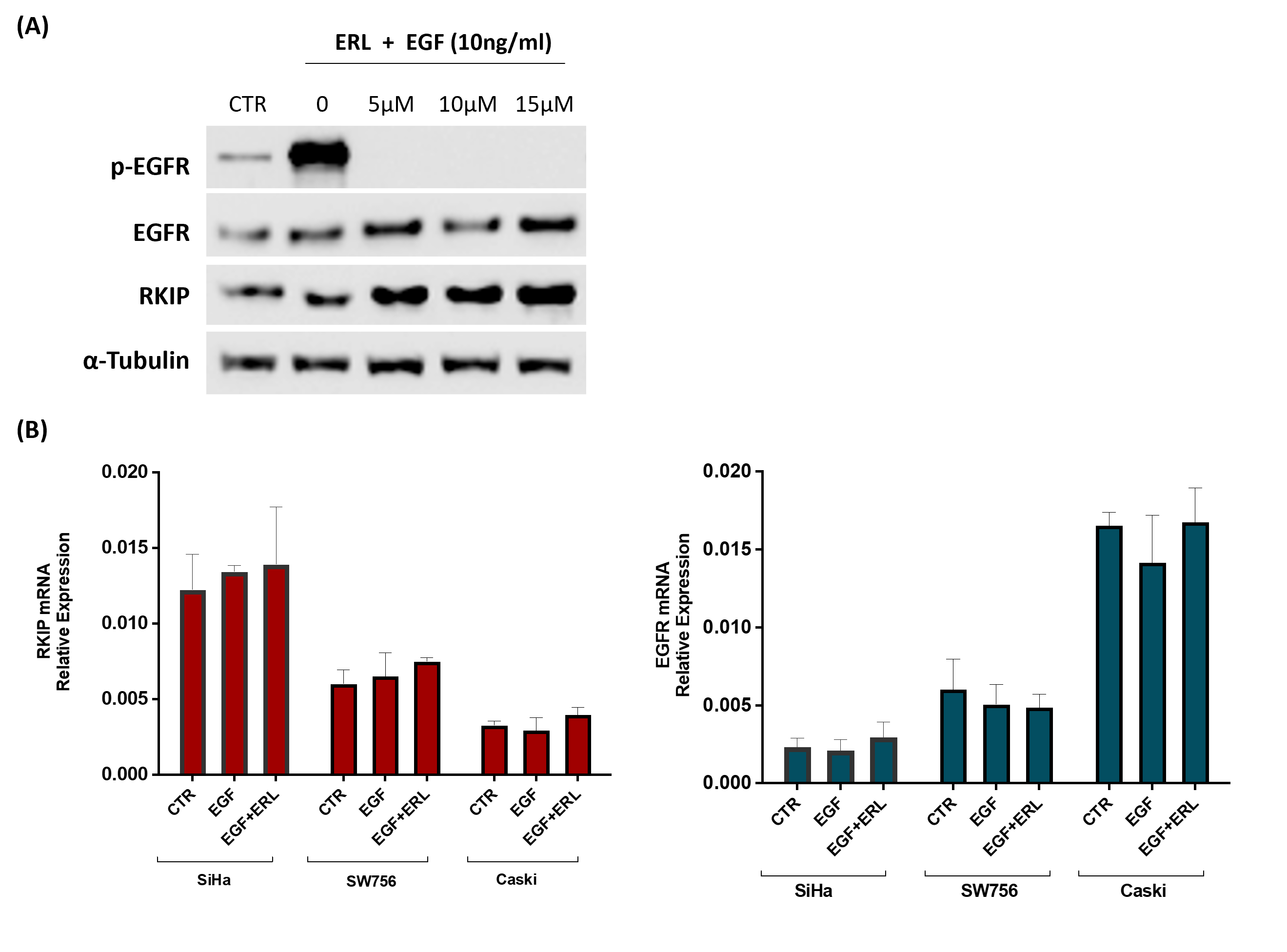

Supplement: Supplementary file 1 [file cancers-16-02182-s001.zip › FigureS3.png]
